# A Scientometric Review of Rasch Measurement: The Rise and Progress of a Specialty

**DOI:** 10.3389/fpsyg.2019.02197

**Published:** 2019-10-22

**Authors:** Vahid Aryadoust, Hannah Ann Hui Tan, Li Ying Ng

**Affiliations:** ^1^National Institute of Education, Nanyang Technological University, Singapore, Singapore; ^2^School of Social Sciences - Psychology, Nanyang Technological University, Singapore, Singapore

**Keywords:** burst, co-citation analysis, Rasch measurement, review, Scientometrics

## Abstract

A recent review of the literature concluded that Rasch measurement is an influential approach in psychometric modeling. Despite the major contributions of Rasch measurement to the growth of scientific research across various fields, there is currently no research on the trends and evolution of Rasch measurement research. The present study used co-citation techniques and a multiple perspectives approach to investigate 5,365 publications on Rasch measurement between 01 January 1972 and 03 May 2019 and their 108,339 unique references downloaded from the Web of Science (WoS). Several methods of network development involving visualization and text-mining were used to analyze these data: author co-citation analysis (ACA), document co-citation analysis (DCA), journal author co-citation analysis (JCA), and keyword analysis. In addition, to investigate the inter-domain trends that link the Rasch measurement specialty to other specialties, we used a dual-map overlay to investigate specialty-to-specialty connections. Influential authors, publications, journals, and keywords were identified. Multiple research frontiers or sub-specialties were detected and the major ones were reviewed, including “visual function questionnaires”, “non-parametric item response theory”, “valid measures (validity)”, “latent class models”, and “many-facet Rasch model”. One of the outstanding patterns identified was the dominance and impact of publications written for general groups of practitioners and researchers. In personal communications, the authors of these publications stressed their mission as being “teachers” who aim to promote Rasch measurement as a conceptual model with real-world applications. Based on these findings, we propose that sociocultural and ethnographic factors have a huge capacity to influence fields of science and should be considered in future investigations of psychometrics and measurement. As the first scientometric review of the Rasch measurement specialty, this study will be of interest to researchers, graduate students, and professors seeking to identify research trends, topics, major publications, and influential scholars.

## Introduction

A recent review of the literature concluded that Rasch measurement is an influential psychometric approach in psychology research (Edelsbrunner and Dablander, 2019). Rasch measurement refers to a family of unidimensional and multidimensional psychometric models inspired by the original formulation of a probabilistic model referred to as “the Rasch model” developed by a Danish mathematician called Georg Rasch in 1957 (Andersen, 1982; Andrich, 1997) to overcome the issues with psychometric testing at that time (Rasch, 1960; Wright and Stone, 1979). At its dawn, the Rasch model was reviewed positively by Coombs (1960), Loevinger (1965), and Sitgreaves (1960) and was embraced and promoted by scholars such as Benjamin Wright (Wright, 1996). In 1960, after learning about Rasch's work from Benjamin Wright, Jimmy Savage invited Rasch to Chicago for a series of lectures from March to June (Wright, 1996; Olsen, 2003). In an interview with David Andrich in May 1979 at Rasch's thatched cottage on the Danish island of Laesoe, Rasch stated:

I lectured on the contents of my book [*Probabilistic Models*.]. Jimmy Savage [Professor of Statistics] started to listen to some of it, but, of course, the mathematical details were so well-known to him. So in the long run, he got tired of it. I did not blame him. So did most of the audience. Only Ben[jamin] [Wright] stayed on. He was regular, took his notes, and I discussed data he brought with him. I also disclosed to him the generalization that Frisch [Nobel-Prize-winning Economist Ragnar] had inspired me to make and the points I was to present at Berkeley [Fourth Symposium on Mathematical Statistics and Probability]. (Andrich, 1997).

Soon after, Rasch (1963) presented the concept of objective measurement through Poisson processes at the International Congress of Psychology. Having a background in physics, Wright who launched courses on Rasch measurement at the University of Chicago in 1964, 1967 and 1968, demonstrated the application of the Rasch model to the Law School Admission Test supporting the argument that the Rasch model is indeed useful in psychometric testing. Another remarkable event was David Andrich's meeting with Georg Rasch in 1972. This marked the start of Rasch's scientific collaborations with Andrich, followed by Andrich's formulation of the rating scale model for polytomous data (Andrich, 1978) that was based on Andersen's (1977) results^1^.

In further two development, the logistic Rasch model that was originally developed to calibrate dichotomous data (x = 0 and 1) (Rasch, 1960; Engelhard, 2012; Engelhard and Wind, 2018), was extended to the partial credit model (Masters, 1982, 1988, 2010), many-facet Rasch measurement (MFRM^2^) (Linacre, 1989), linear logistic test model (Fischer, 1995), linear rating scale (Fischer and Parzer, 1991) and partial credit models (Fischer and Ponocny, 1994), linear and repeated measures models (Hoijtink, 1995), extended rating scale and expansions of the partial credit model for assessing change (Fischer and Parzer, 1991; Fischer and Ponocny, 1994), and the mixture distribution Rasch model (Rost, 1990; von Davier, 1996), to name a few. There were also a number of mixture distribution Rasch models, such as loglinear multivariate mixture Rasch models (Kelderman, 2007) and mixture hybrid models (von Davier and Yamamoto, 2007). Scholars modified the Rasch multidimensionality^3^ (Embretson, 1991; Ackerman, 1994), such as the multidimensional random coefficients multinomial logit (MRCML) model (Adams et al., 1997; Wang et al., 1997; Wu et al., 1998; for further developments of the model, please see e.g., Kelderman and Rijkes, 1994; Fischer and Molenaar, 1995; Briggs and Wilson, 2003).

Fischer (2010)—who had been a regular attendant at Rasch's office hours—wrote that after spending 2 months in Copenhagen with Rasch, he developed a computer software for conditional maximum likelihood estimation of the Rasch model in 1967, which was used extensively in German-speaking countries. In 1983, Benjamin Wright and Mike Linacre developed Microsale – the first Rasch software that was robust against missing data. Microscale was further developed into Bigscale in 1989, Bigsteps in 1991, and Winsteps in 1998 (Linacre, 1998, 2004). In addition to these computer programs, many other Rasch computer programs had been developed. For example, the first programs with wide usage were independently published around 1967 by Benjamin Wright and Gerhard Fischer, and the first Rasch software to be used for large-scale data analysis was BICAL^4^ (Wright et al., 1978). Some of the most commonly used Rasch measurement computer programs commercially available today include RUMM2030 (Andrich et al., 2009), ACER ConQuest (Wu et al., 2007), Winmira (von Davier, 2001), Facets and Winsteps (Linacre, 2019a,b; a list of Rasch software is available from: https://www.rasch.org/software.htm).

The method proposed by Georg Rasch for calibrating test items was preceded by Thorndike's (1919) and Thurstone's (1925) scaling methods which, like the Rasch models, were concerned with converting raw scores to scale-free measures of ability. According to Engelhard (1984, p. 26), the three approaches were similar in terms of “[t]he[ir] concept of an underlying latent trait [that] plays a central role in the quest for invariance with the three scaling methods.” While Thorndike's (1919) and Thurstone's (1925) scaling methods assumed a normal distribution for test data, Rasch's approach did not assume normality as it was set out to create scale free measures at the individual rather than group level of analysis (Engelhard, 1984). The normality assumption also underlies Birnbaum's (1967), Lord's (1952), and Lord and Novick's (1968) latent trait and item response theory (IRT) models, all of which strongly influenced educational and psychological measurement.

Finally, the enormous potential of Rasch models is recognized in various fields, including health and medical research (Tennant et al., 2004b; Pallant and Bailey, 2005; Tennant and Conaghan, 2007; e.g., Belvedere and de Morton, 2010; Sica da Rocha et al., 2013), assessment of educational and language skills (e.g., McNamara and Knoch, 2012; Eckes, 2015), rater training (Engelhard and Wind, 2018), and psychological measurement (e.g., Bond and Fox, 2015), to name a few.

## The Present Study

There are reviews of the Rasch measurement specialty, such as Smith's (2019) and Wright's (1996) lists of key events, Olsen's (2003) extensive PhD thesis on the life and contributions of Georg Rasch, Bond's (2005) historical review of Rasch measurement, Panayides et al.'s (2010) historical account and defense of Rasch measurement in England, Belvedere and de Morton's (2010) review of studies validating mobility scales, Tesio et al.'s (2007) review of the application of Rasch measurement in rehabilitation research, Fischer's (2010) historical review of Rasch measurement in Europe, Wright's (1997) review of measurement in social sciences, and McNamara and Knoch's (2012) review of Rasch measurement in language assessment. In contrast to these positive accounts, Goldstein and Wood's (1989, p. 139) review of IRT (Rasch included) asserted that research in this field had shown a “disappointing lack of advance” in its 50 years of existence (see also Chien and Shao, 2019).

Several textbooks on Rasch measurement also reviewed the history and evolution of the speciality, highlighting the parts that were more relevant to the theme of the books (e.g., Andrich, 1989; Engelhard and Wind, 2018). In our view, to characterize the Rasch measurement specialty, it is important to determine the forces that have shaped its evolution over the years. These forces include impactful research trends, influential researchers, publications, and research outlets where the results of investigations have been published (Chen, 2016b). In addition, it is important to investigate the inter-domain trends through which the Rasch measurement specialty is linked to other fields of science.

To achieve this objective, we used a co-citation technique to identify the main players in the field. This method contrasts with previous investigations that were descriptive in nature (Tennant, 2011; Edelsbrunner and Dablander, 2019). In the present paper, we adopted advanced visualization (Chen et al., 2010) and text-mining methods. The text-mining methods include (i) author co-citation analysis (ACA) (Leydesdorff, 2005; Zhao and Strotmann, 2008), which considers two authors to be co-cited if they are cited together in a paper; (ii) document co-citation analysis (DCA) (e.g., Chen, 2004, 2006; Chen et al., 2008), where a co-citation instance occurs when two sources are cited together in one paper; (iii) journal co-citation analysis (JCA), which is used to identify journals cited together in one paper; and (iv) keyword analysis, where instances of two keywords appearing together are analyzed (Chen, 2017). These methods are similar to “big data” techniques suitable for computationally analyzing extremely large datasets to reveal active or developing research trends consisting of nodes with citation bursts defined as a publication's rapid increase in citation and influence (Chen, 2017). To investigate the inter-domain trends that link the Rasch measurement specialty to other specialties, we used a dual-map overlay (Chen and Leydesdorff, 2014) to investigate specialty-to-specialty connections (see *Methodology* for further details).

The co-citation technique, compared with methods such as narrative reviews (Chen et al., 2010), offers several main benefits such as: (i) the use of extensive bibliographic data adopted from Web of Science (WoS) and/or Scopus; (ii) reducing the inconvenience of analyzing huge datasets and providing computation of co-citations; (iii) leveraging computer programs for visualization and text-mining, such as CiteSpace (Chen et al., 2010; Chen, 2016a); and (iv) allowing researchers to produce a quantitative interpretation of the past and present state of specialties.

The research questions of the present study are as follows:

1. Where is Rasch measurement research situated on the map of the WoS and how is it linked with other research fields?
2. What are the impactful publications (bursts), major research trends, and keywords? What does the content of the major research trends reveal about the Rasch measurement specialty?

## Methodology

### Data Source

The data used for analyses comprised 5,365 publications on theory and practice in Rasch measurement between 01 January 1972^5^ and 03 May 2019 with their 108,339 unique references downloaded from the WoS. These included 49,991 citing articles (that cited one or more publications in the corpus; articles without self-citations = 45,765). The reason for choosing WoS over other databases such as Scopus was its scope and coverage of published research. As our search of Scopus returned a smaller sample (<4,000 publications), we decided to adopt the database from WoS. However, a caveat is that the cited references in the database from WoS only included the first author^6^; Other contributors to the publications were thus not included in the analysis.

**Figure 1:**
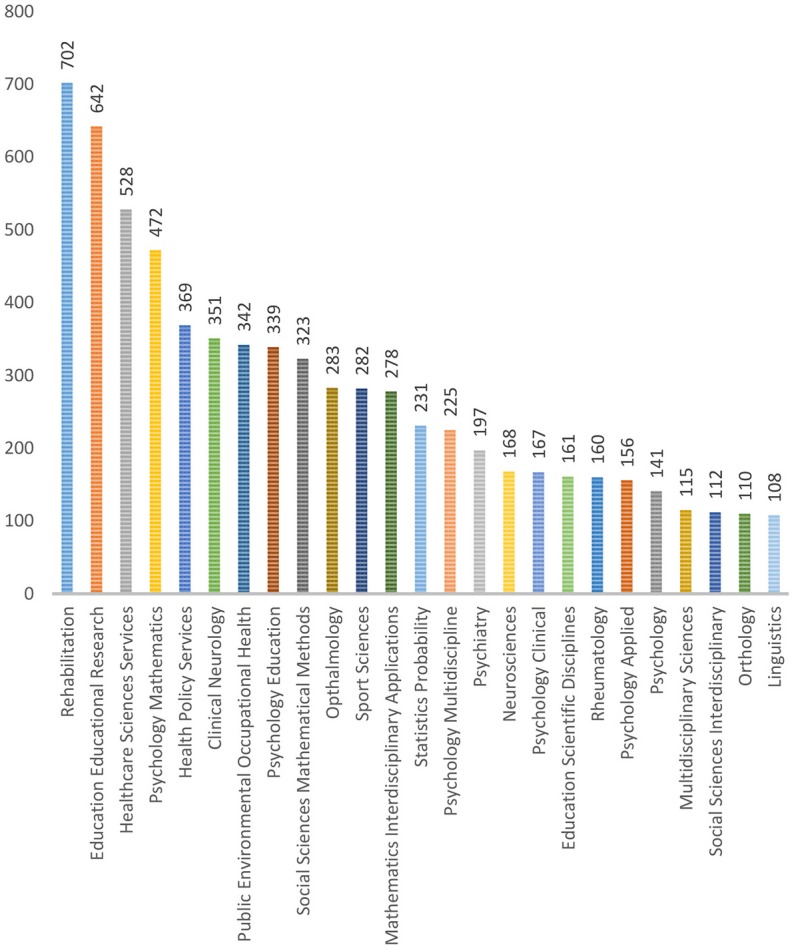
Frequency of publications using Rasch measurement in different fields (Source: WoS).

The search code used was “*TOPIC: (“Rasch model”) OR TOPIC: (“Rasch measurement”) OR TOPIC****:****(“Rasch analysis”)*.” Figure 1 presents the descriptive statistics of the dataset downloaded from WoS. Rehabilitation (13.09%), education and educational research (11.97%), health care sciences services (9.84%), psychology / mathematics (8.80%), and health policy services (6.88%) constitute the top five disciplines with the highest application of the Rasch model. By contrast, linguistics (2.01%), orthopedics (2.05%), social sciences interdisciplinary (2.09%), multidisciplinary sciences (2.14%), and psychology (2.63%) form the bottom five adopters of the Rasch model. Each field can be further broken down into subfields. For example, in linguistics, which is the smallest field identified, U Koch (publications = 6), V Aryadoust (publications = 5), G Janssen (publications = 5), and J Tarace (publications = 5) achieved the highest number of publications among scholars in this field.

### Research Question 1: The Dual-Map Overlay

To answer research question one, we used CiteSpace (Chen, 2016a) to generate a dual-map overlay that displayed a first base map of citing journals and a second base map of cited journals in the same user interface by using the influential journals of all fields retrieved from WoS to generate the overlay (Chen and Leydesdorff, 2014). Trajectories were then generated from the citing journals and cited journals to provide a better overview of the citations. Subsequently, the Blondel algorithm (Leydesdorff et al., 2013) was used to assign these journals to a cluster. This algorithm provided access to community networks of varying resolutions of community detection by finding high modularity partitions and unfolding a full hierarchical community layout (Blondel et al., 2008). The dual-map overlay allowed us to conduct several visual analyses as we were able to see the sources and targets of citations from various publications and the distributions of the citation arcs. This allowed us to investigate inter-specialty relationships and citation patterns from a group of publications.

### Research Question 2: Bursts, Trends, and Their Influence

To answer the second research question, we adopted Chen et al.'s (2010, p. 1,389) “multiple-perspective co-citation analysis” technique that comprised the analysis of “structural, temporal, and semantic patterns as well as the use of both citing and cited items for interpreting the nature of co-citation clusters.” The components of this approach are discussed below.

#### Selection of Nodes

The two most recommended methods for node selection are Top *N* and Top *N%*. The Top *N* per slice procedure used in this study, selected the most cited items from each slice to form a network, according to the input value and node type determined by the user. We chose a value of 50 and multiple node types, so the top 50 most cited items were displayed and ranked accordingly. The Top *N%* per slice procedure displayed the percentage of most cited items according to a value determined by the user.

#### Network Development

The WoS dataset was used to construct co-citation networks for authors and publications (see below) for network development. Following Chen et al. (2010), ACA, DCA, JCA, and keyword analysis were performed to cluster co-citing authors (White and McCain, 1998; Chen, 1999; Leydesdorff, 2005; Zhao and Strotmann, 2008), co-citing publications, journals, and keywords (Small and Sweeney, 1985; Small and Greenlee, 1986; Chen, 2004, 2006; Chen et al., 2008), respectively.

Temporal and structural metrics were adopted to investigate network and cluster properties. Citation burstness and sigma (Σ) are temporal metrics. Knowing whether the citation count of a particular reference rose and when the rise occurred was important for citation analysis. Burst detection determined if the fluctuations for a specific frequency function, within a time period were significant. Sigma – the combination of betweenness centrality and burstness, was calculated as *(centrality*+*1)*^*burstness*^ (Chen et al., 2010). This metric was used to identify and measure novel ideas presented in scientific publications (Chen et al., 2009). Sigma ranged from 0 to 1. Case studies by Chen et al. (2009) showed that the highest sigma values were usually associated with Nobel Prize and other award-winning researchers.

The average silhouette score (Rousseeuw, 1987), modularity Q (Newman, 2006), and betweenness centrality (Freeman, 1977) are structural metrics. The silhouette metric estimates the level of uncertainty when interpreting a cluster's nature. The silhouette value ranges from −1 to 1; a value of 1 suggests that a cluster is distinct from other clusters. The modularity Q ranges from 0 to 1 and measures the extent to which a network can be divided into modules. A high modularity score suggests a network with divisible structure, while a low modularity score suggest less distinct separation between clusters. The extent to which a node connects other nodes in a network is measured by the betweenness centrality metric. Scientific publications with high betweenness centrality values indicate potentially revolutionary material. These metrics are thus useful for finding influential scientific publications.

#### Visualization and Labeling of Clusters

We used the multidimensional clustering method for identification of clusters and their connections. We used two visualization methods to demonstrate the shape and form of the networks: the timeline view and the cluster view. The timeline view consisted of a range of vertical lines that represent time zones chronologically arranged from the left to right side (Chen, 2014). In this view, while the horizontal arrangement of nodes were restricted to the time zones they are located on, the nodes were allowed to have vertical links with nodes in other time zones (Chen, 2014). The cluster view, on the other hand, produced spatial network representations that were color-coded and automatically labeled in a landscape format.

We further used the log-likelihood ratio (LLR) method for automatic extraction of cluster labels. This method was found to provide the best results in terms of uniqueness and coverage (Dunning, 1993; Chen, 2014). Although the latent semantic indexing (LSI) and mutual information (MI) methods were available, they were not used in this study as their precision was lower compared with LLR (Chen, 2014).

## Results

### Dual-Map Overlay

**Figure 2:**
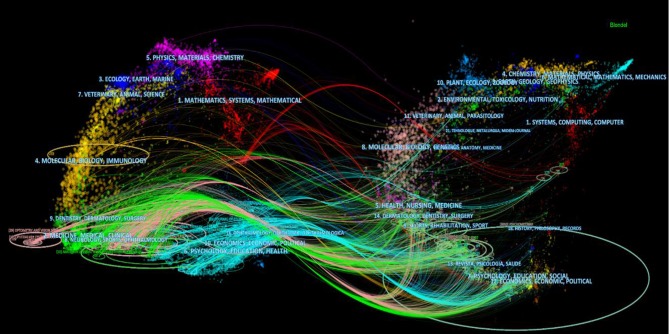
The dual-map overlay of the Rasch measurement specialty generated by CiteSpace (Chen, 2016a).

Figure 2 shows the generated dual-map overlay: the citing journals are on the left, the cited journals are on the right, and the citation links tells us what journal the citing journal cited from. The trajectory of the citation links provides an understanding of inter-specialty relationships. A shift in trajectory from one region to another would indicate that a discipline was influenced by articles from another discipline. It was evident that medicine, sports, ophthalmology, neurology, and psychology were the dominant fields at the start of the trajectory. In contrast, health, nursing, sports, psychology, and economics dominated the end of the trajectory.

### Author Co-citation Analysis (ACA)

The modularity Q score of the ACA network was 0.5411. As the modularity Q score represented how well the network was split into various independent clusters (Chen et al., 2010), a score of 0.5411 suggested that the networks and clusters were moderately well-structured. However, the boundaries that separated the clusters were not definitive. The average silhouette score was 0.3225, suggesting that the cluster had respectable heterogeneity. Table 1 presents the top 15, middle 5, and lowest 5 ranking publications as sample author bursts computed via ACA (for a complete list, please see Appendix 1). The author with the highest burst strength was WJ Boone (strength = 43.9175), whose burstness started in 2016 and continued to grow in 2019, followed by G Engelhard (strength = 42.3541, 2016–2019) and H Kelderman (strength = 32.5274, 1988–2005). Within the stipulated timeframe, the rapid changes in the number of citations received reflected the growing importance of the authors' papers and ideas.

**Table 1:** Sample author bursts computed via author co-citation analysis (ACA).

**Cited authors**	**Burst strength**	**Begin**	**End**	**Span**
Boone WJ	43.9175	2016	2019	3
Engelhard G	42.3541	2016	2019	3
Kelderman H	32.5274	1988	2005	17
Bock RD	32.2103	1983	2007	24
Lamoreux EL	31.5968	2008	2013	5
Lord FM	31.2723	1977	2004	27
Hu LT	31.2241	2013	2019	6
Christensen KB	30.9013	2016	2019	3
Hagquist C	30.5041	2012	2019	7
Messick S	30.2655	2014	2019	5
Molenaar IW	30.1958	1986	2005	19
Samejima F	29.5008	1982	2006	24
Velozo CA	29.2273	2004	2009	5
Hobart J	28.1438	2012	2019	7
Lincare MJ	31.7059	2014	2019	5
Drasgow F	11.0877	1993	2003	10
Rosenbaum PR	10.7626	1990	2005	15
Mokken RJ	10.7626	1990	2005	15
Anderson EB	10.5197	1998	2002	4
Baker FB	10.4419	2010	2012	2
Mccullagh P	4.4323	1993	2003	10
Follmann D	4.3777	1993	1998	5
Zhu WM	4.3118	1996	2001	5
Tatsuoka KK	4.2288	1995	2001	6
Haley SM	4.1974	2010	2011	1

*Modularity Q = 0.5411; average silhouette = 0.3225*.

As indicated on Table 1, SM Haley had the smallest burst (strength = 4.1974), whose burst lasted from 2010 to 2011. Notably, FM Lord had the longest lasting burst with a span of 27 years (strength = 31.2723). In contrast, SM Haley along with 12 other authors only had a burst span of one year (see Appendix 1 for a comprehensive list of chronologically ordered bursts).

### Document Co-citation Analysis (DCA)

**Figure 3:**
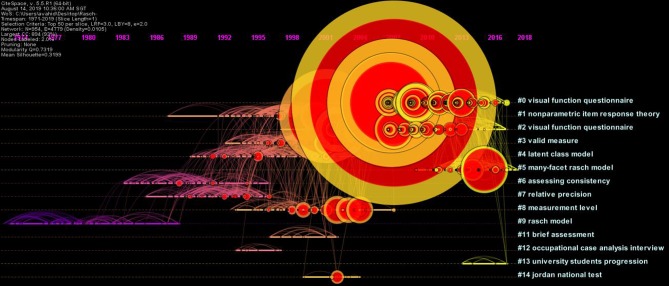
Timeline view of the document co-citation analysis (DCA) network generated by CiteSpace (Chen, 2016a) (Modularity Q = 0.7319; average silhouette = 0.3199).

**Figure 4:**
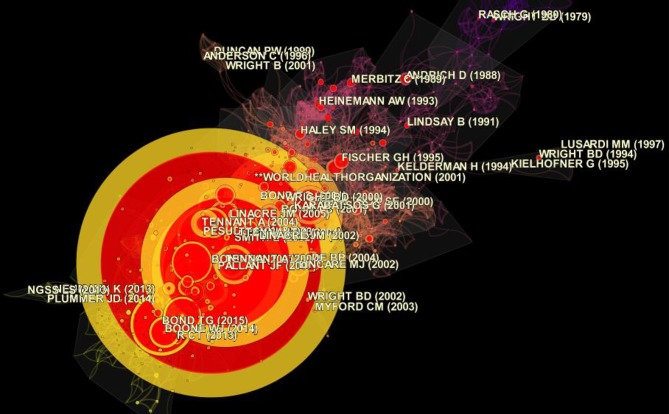
Cluster view of the document co-citation analysis (DCA) network generated by CiteSpace (Chen, 2016a) (modularity Q = 0.7319; average silhouette = 0.3199).

The timeline view and cluster view of DCA were generated to gain a clearer understanding of the occurrences and magnitudes of the bursts of different publications in the different clusters (Figures 3, 4). The clusters were numbered and ranked in terms of their size, with cluster #0 being the largest cluster. The size of the circle reflected the magnitude of the publication's influence: the larger circle, the higher the number of citations. The burstness of the author's publication was indicated by the red tree rings. From the DCA analysis, 57 clusters emerged. Figure 3 shows the top 14 largest clusters. Cluster #0 on visual questionnaires, was the largest cluster and showed activity from 2003 until the present. The presence of large nodes and nodes with the red tree rings in this cluster indicated that many publications from this cluster were highly influential or had high citation bursts. The size of the cluster was 174, and this accounted for 18.26% of all clusters. Cluster #1 on non-parametric item response theory, was the second largest cluster with a size of 114 (11.96%), followed by cluster #2, on visual function questionnaire, with a size of 103 (10.81%). Cluster #3 on valid measure (validity), cluster #4 on latent class model, and cluster #5 on many-facet Rasch model had sizes of 97 (10.17%), 80 (8.39%), and 75 (7.87%) respectively. The cluster view of the DCA network is presented in Figure 4. The cluster labels were turned off for clarity and only the authors' name of some influential publications were displayed.

The top three largest clusters show activity for a span of roughly 20 years each. By contrast, the smaller clusters had shorter spans of 4–5 years each. The clusters had distinctive division of modules and respectable heterogeneity, with modularity Q score and average silhouette score at 0.7319 and 0.3199 respectively.

Table 2 presents the sample document bursts that were computed via DCA (see Appendix 2 for a comprehensive list of chronologically ordered bursts). Publications with high levels of strength signify major milestones in the development of Rasch measurement. Two notable publications by TG Bond (strength = 133.0131 and 88.1181, respectively) had the largest magnitudes of document bursts, with spans lasting 5 and 6 years, respectively. This suggested that works by TG Bond were not only highly influential but had greatly contributed to the development of Rasch measurement. A Tennant's publication (strength = 83.7317) also had high strength document burst with a span of 4 years. At the other end, the citation burst of the lowest magnitude was EB Andersen (strength = 4.1214) with a burst span of 2 years. Several authors, such as A Tennant, JM Linacre, K Pesudovs, and WJ van der Linden, shared the longest spanning document bursts at 7 years.

**Table 2:** Sample document bursts computed via document co-citation analysis (DCA).

**First authors**	**Year**	**Strength**	**Begin**	**End**	**Span**	**References**
Bond TG, Applying the Rasch Model: Fundamental Measurement in the Human Sciences 2nd Edition	2007	133.0131	2010	2015	5	Bond and Fox, 2007
Bond TG, Applying the Rasch Model: Fundamental Measurement in the Human Sciences 1st Edition	2001	88.1181	2003	2009	6	Bond and Fox, 2001
Tennant A, Arthritis & Rheumatism-Arthritis Care & Research	2007	83.7317	2011	2015	4	Tennant and Conaghan, 2007
Bond TG, Applying the Rasch Model: Fundamental Measurement in the Human Sciences 3rd Edition	2015	57.9267	2016	2019	3	Bond and Fox, 2015
Pallant JF, British Journal of Clinical Psychology, V46, P1	2007	52.4344	2009	2015	6	Pallant and Tennant, 2007
Smith E, Journal of Applied Measurement, V3, P205	2002	49.3961	2006	2010	4	Smith, 2002
Embretson SE, Item Response Theory	2000	33.1571	2003	2008	5	Embretson and Reise, 2000
Hagquist C, International Journal of Nursing Studies, V46, P380	2009	32.1003	2012	2017	5	Hagquist et al., 2009
Engelhard G, Invariant Measurement: Using Rasch Models in the Social, Behavioral, and Health Sciences	2013	30.8451	2016	2019	3	Engelhard, 2013
Linacre JM, Journal of Applied Measurement, V3, P85	2002	28.0657	2006	2010	4	Linacre, 2002
Hobart J, Health Technology Assessment, V13, P1	2009	27.8369	2012	2017	5	Hobart and Cano, 2009
Tennant A, Rasch measurement transactions, V20, P1048	2006	27.8189	2008	2014	6	Tennant and Pallant, 2006
Tennant A, Value in Health, V7	2004	27.3566	2006	2012	6	Tennant et al., 2004a
Boone WJ, Rasch Analysis in the Human Sciences	2013	26.274	2016	2019	3	Boone et al., 2013
World Health Organization, International Classification of Functioning, Disability and Health (ICF)	2001	25.7576	2004	2009	5	
Pesudovs K, Investigative Ophthalmology & Visual Science, V44, P2892	2003	21.0882	2004	2011	7	Pesudovs et al., 2003
van der Linden WJ, Handbook of Modern Item Response Theory	1997	18.5703	1998	2005	7	van der Linden and Hambleton, 1997
Tennant A, Medical Care, V42, P37	2004	20.8859	2005	2012	7	Tennant et al., 2004b
Linacre JM, A user's guide to Winsteps-Ministep: Rasch-model computer programs. Program manual 3.68.	2009	16.7212	2010	2017	7	Linacre, 2009
Boone WJ, Science Education, V95, P258	2011	8.4305	2014	2017	3	Boone et al., 2011
Pesudovs K, Optometry and Vision Science, V87, P285	2010	8.2228	2011	2016	5	Pesudovs, 2010
Mangione CM, Archives of Ophthalmology, V119, P1050	2001	8.1752	2005	2008	3	Mangione et al., 2001
Raîche G, Rash Measurement Theory, V19, P1012	2005	8.1466	2012	2013	1	Raîche, 2005
Lindsay B, Journal of the American Statistical Association, V86, P96	1991	8.0526	1993	1999	6	Lindsay et al., 1991
Velozo CA, Archives of Physical Medicine and Rehabilitation, V76, P705	1995	4.2197	2000	2003	3	Velozo et al., 1995
Klauer KC, Rasch Models (Book chapter), PP97-110	1995	4.2197	2000	2003	3	Klauer, 1995
Linacre JM, A user's guide to Facets Rasch-model computer programs	2014	4.1469	2016	2019	3	Linacre, 2014
Smith E, Journal of Applied Measurement, V1, P303	2000	4.1466	2004	2006	2	Smith, 2000
Andersen EB, British Journal of Mathematical and Statistical Psychology, V26, P31	1973	4.1214	1979	1981	2	
Tennant A, The British Journal Of Rheumatology, V35, P574	1996	6.3264	1997	2004	7	Tennant et al., 1996

*Modularity Q = 0.7319; average silhouette = 0.3199*.

Table 3 shows the publications with high centrality and high sigma. As mentioned above, publications with high centrality scores were highly influential and items with high sigma scores indicate scientific novelty. A Tennant's publication (2007) was both highly influential and contained potentially scientific novel revelations (centrality = 0.15; sigma = 156925.6), followed by van der Linden and Hambleton (1997) (centrality = 0.15; sigma = 13.5) and two editions of Bond and Fox's (2001, 2007) monograph on Rasch measurement (centrality = 0.03 and sigma = 17.88; centrality = 0.09 and sigma = 88484.75, respectively) (see Appendix 1 for a comprehensive list of chronologically ordered bursts). High betweenness centrality indicated that the publications in Table 3 connected two or more clusters and, therefore, two or more themes (clusters) (Chen et al., 2009, 2010). As they connected different themes, they were also likely to be a synthesis of different ideas into a new one, and could be revolutionary in providing this connection. As previously stated, higher sigma values indicated the novelty of these publications (Chen et al., 2009; Chen, 2017).

**Table 3:** Centrality and sigma estimated via document co-citation analysis (DCA).

**Centrality**	**First authors**	**Cluster #**	**References**
0.15	Tennant A, 2007, Arthritis & Rheumatism-Arthritis Care & Research, 57, 1358	0	Tennant and Conaghan, 2007
0.15	van der Linden WJ, 1997, Handbook of modern item response theory	1	van der Linden and Hambleton, 1997
0.14	Adams RJ, 1997, Applied Psychological Measurement 21, 1	1	Adams et al., 1997
0.14	McHorney CA, 1997, Annals of Internal Medicine, 127, 743	3	McHorney, 1997
0.13	Fischer GH, 1995, Rasch models: foundations, recent developments, and applications.	4	Fischer, 1995
0.11	Karabatsos G, 2001, Journal of Applied Measurement, 2, 389	4	Karabatsos, 2001
0.1	de Boeck P, 2004, Explanatory item response models	1	de Boeck and Wilson, 2004
0.1	Fischer GH, 1987, Psychometrika, 52, 565	9	
0.09	Bond TG, 2007, Applying the Rasch model: Fundamental measurement in the human sciences (2nd ed.)	0	Bond and Fox, 2007
0.09	Hobart J, 2009, Health Technology Assessment, 13, 1	0	Hobart and Cano, 2009
**Sigma**			
156925.6	Tennant A, 2007, Arthritis & Rheumatism-Arthritis Care & Research, 57, 1358	0	Tennant and Conaghan, 2007
88484.75	Bond TG, 2007, Applying the Rasch model: Fundamental measurement in the human sciences (2nd ed.)	0	Bond and Fox, 2007
17.88	Bond TG, 2001, Applying the Rasch model: Fundamental measurement in the human sciences (1st ed.)	1	Bond and Fox, 2001
13.5	Van der Linden, 1997, Handbook of modern item response theory	1	van der Linden and Hambleton, 1997
11.15	Hobart J, 2009, Health Technology Assessment, 13, 1	0	Hobart and Cano, 2009
10.72	Hagquist C, 2009, International Journal of Nursing Studies, 46, 380	0	Hagquist et al., 2009
9.86	Fischer GH, 1995, Rasch models: foundations, recent developments, and applications	4	Fischer, 1995
7.66	Embretson SE, 2000, Multivariate Applications Books Series. Item response theory for psychologists	1	Embretson and Reise, 2000
5.84	Adams RJ, 1997, Applied Psychological Measurement, 21, 1	1	Adams et al., 1997
3.6	Andrich D, 1988, Rasch models for measurement. Series: Quantitative applications in the social sciences	6	Andrich, 1988

*Modularity Q = 0.7319; average silhouette = 0.3199*.

### Journal Co-citation Analysis (JCA)

Table 4 demonstrates sample journal bursts computed via JCA. A modularity Q score of 0.4626 suggested that the JCA network and clusters were moderately well-structured. However, the boundaries that delineated the clusters were not clear. The average silhouette score was 0.3522, suggesting that the cluster had respectable heterogeneity. The journal with the largest burst was *PLoS ONE* (strength = 83.752, 2015–2019), and the burstness was still growing. *Arthritis and Rheumatism-Arthritis Care and Research* (strength = 80.62, 2009–2015) had the second largest burst, followed by the *Journal of Outcome Measurement* (strength = 74.264, 1998–2007). The *International Journal of Biometrics* (strength = 9.6156), which had the longest burst span of 24 years from 1982 to 2006. Eight journals, including *Applied Measurement in Education* (strength = 7.073, 2009–2010), had a burst span of one year (see Appendix 3 for a comprehensive list of chronologically ordered bursts). Notably, the *Journal of Outcome Measurement* was a predecessor of *Journal of Applied Measurement (JAM)*. Thus, its impact was combined with that of *JAM* (strength = 62.4672).

**Table 4:** Sample publication bursts computed via journal co-citation analysis (JCA).

**Cited journals**	**Strength**	**Begin**	**End**	**Span**
PLoS ONE	83.7516	2015	2019	4
Arthritis & Rheumatism-Arthritis Care & Research	80.6204	2009	2015	6
Journal of Outcome Measurement	74.2638	1998	2007	9
Psychometrika	68.4709	1976	1999	23
Journal of Applied Measurement	62.4672	2005	2010	5
Medical Care	59.4932	2004	2012	8
Thesis Eleven: Critical Theory and Historical Sociology	53.7951	2016	2019	3
British Journal of Clinical Psychology	49.9896	2009	2015	6
Introduction to Rasch Measurement: Theory, Models, and Applications (Book)	44.6339	2006	2012	6
Health and Quality of Life Outcomes	43.4422	2016	2019	3
Item Response Theory (Book)	40.6607	2003	2008	5
Applied Psychological Measurement	36.9212	1983	2000	17
Rasch Models (Book)	33.6824	1996	2003	7
Applying the Rasch Model (Book)	32.4572	2003	2009	6
International Journal of Nursing Studies	32.0875	2012	2017	5
Annals of Internal Medicine	13.6894	1997	2004	7
Clinical Rehabilitation	13.52	2002	2011	9
Stroke	13.1618	2002	2009	7
Neurology	12.9825	2010	2013	3
Journal of Statistical Software	12.7044	2017	2019	2
International Journal of Biometrics	9.6156	1982	2006	24
Applied Measurement in Education	7.073	2009	2010	1
Review of Educational Research	4.3638	1980	1999	19
Educational and Psychological Measurement	4.3176	1977	1981	4
Educational Research Methodology and Measurement	4.2394	2002	2005	3
Zeitschrift fur Experimentelle und Angewandte Psychologie	4.1393	1981	1983	2
Conditional Inference	4.137	1979	1981	2

*JCA has also identified influential books. Modularity Q = 0.4626; average silhouette = 0.3522*.

### Keywords Co-citation Analysis

Keywords with high strength indicated influential ideas that originate from the clusters (Modularity Q = 0.406; average silhouette = 0.5166). Table 5 shows sample keyword bursts computed via keyword analysis. The keyword with the burst of the largest magnitude was “surveys and questionnaire(s)” (strength = 94.7513), with a burst span of 4 years, and it is still growing. Next were “psychology” (strength = 58.3953, 2014–2019) and “procedure” (strength = 48.8441, 2014–2019). The keyword “personality inventory” was not only the smallest burst, it also had a burst span of one year (strength = 9.9687). The longest burst spans of 20 years were “comparative study” (strength = 6.694, 1986–2006) and “clinical article” (strength = 8.201, 1986–2006).

**Table 5:** Sample keyword bursts computed via co-occurring author keywords analysis and keyword plus.

**Keywords**	**Strength**	**Begin**	**End**	**Span**
Surveys and Questionnaire	94.7513	2015	2019	4
Psychology	58.3953	2014	2019	5
Procedure	48.8441	2014	2019	5
United States	28.3694	1997	2009	12
Statistics and Numerical Data	28.276	2014	2017	3
Functional Assessment	26.7444	1996	2008	12
Elderly	26.0642	2014	2017	3
Reproducibility	25.1707	2014	2017	3
Patient Reported Outcome	24.2858	2017	2019	2
Statistical Model	23.6862	1998	2005	7
Factor Analysis	23.127	2016	2019	3
Algorithm	20.3911	2012	2016	4
Methodology	19.8192	2010	2013	3
Scoring System	19.6275	2002	2009	7
Test Retest Reliability	18.1344	2017	2019	2
Rash Model	10.3259	1989	1996	7
Health Status	9.9187	2001	2009	8
Validation Process	9.6254	2017	2019	2
Education	9.3478	2014	2017	3
Rasch Modeling	9.2283	2014	2016	2
Clinical Article	8.201	1986	2006	20
Comparative Study	6.694	1986	2006	20
Observer Variation	4.0297	1996	1998	2
Software	3.9907	1997	1999	2
Computer Program	3.9907	1997	1999	2
Outcomes Research	3.9689	2001	2002	1
Personality Inventory	3.9687	2000	2001	1

*Modularity Q = 0.406; average silhouette = 0.5166*.

## Discussion

The present study adopted several co-citation techniques to investigate published research that applied or discussed Rasch measurement theory. The findings and research questions are discussed in this section.

### First Research Question

To address the first research question on where Rasch measurement was located on the map of WoS and how it was linked to other research fields, a dual-map overlay was generated. As shown in the Results section, certain fields were especially prominent, such as medicine, neurology, and psychology, indicating that the Rasch models were commonly used in such fields. The left and the right base maps also had some common fields such as molecular biology and immunology on the left, and molecular biology and genetics on the right. Some of these fields were connected to each other as well. This was indicative that reciprocal citations within the field using Rasch models were common. To develop a full picture of the application of Rasch measurement in each field, additional intra-disciplinary studies will be needed to investigate how the model has contributed to research in each field.

### Second Research Question

#### Influential Authors, Documents, and Journals

To address the second research question, a large database consisting of 5,365 publications on theory and practice in Rasch measurement between 01 January 1972 and 03 May 2019 and their 108,339 unique references were analyzed. We used a multiple perspectives approach consisting of network visualization methods, ACA, DCA, JCA, and keyword analysis to generate different networks and investigate the different dimensions of the Rasch measurement specialty. The top authors with the highest bursts included Boone WJ, Engelhard G, Kelderman H, Bock RD, Lamoreux EL, Lord FM, Hu LT, Christensen KB, Hagquist C, and Messick S (see the relevant tables and Appendix). Examining the areas of expertise of these authors revealed backgrounds not only in the Rasch models (e.g., Boone WJ and Engelhard G) but also in IRT models (e.g., Bock RD and Lord FM), and structural equation modeling (e.g., Hu LT), indicating the links between these fields and perhaps the influence that the Rasch measurement field has received from other fields (see section **Limitations** for a further discussion).

The most influential publication in Rasch measurement was the volume “*Applying the Rasch Model: Fundamental Measurement in the Human Sciences*” by Bond and Fox (2007), followed by its earlier edition (Bond and Fox, 2001). The third edition of the book ranks fourth after Tennant and Conaghan's (2007) article in *Arthritis and Rheumatism-Arthritis Care and Research*. From this perspective, Bond and Fox's book was exceptional as its three editions were among the top four influential publications in the Rasch measurement field.

Impactful journals include *PLoS ONE* and *Arthritis* & *Rheumatism-Arthritis Care and Research*, followed by *Journal of Outcome Measurement (JOM), Psychometrika*, and *Journal of Applied Measurement (JAM)*. According to JAM Press (2018), “JOM was the predecessor of JAM and contains many articles related to Rasch measurement in education and the health sciences.” Lastly, impactful keywords included surveys, questionnaires, and psychology. Although there was no document from a journal or book by an author that had dominated the top of all lists, there were generally links where the most impactful authors had publications among the top 20 most impactful documents. This applied to comparisons of journals and authors as well as documents and journals, where the most influential authors published in the most influential journals. This indicated that there was a concentration of influence. Perhaps the Rasch measurement specialty was not extensively used beyond the fields in which those authors and journals were published. This hypothesis may be examined in future research.

Another general observation was the increase in number of fields that adopted the model over the years. Despite several decades after the publication of Rasch's (1960) book, the model was unknown to many fields. According to Fischer (2019, personal communication), “in 1970 there existed worldwide only four centers where research on the RM [Rasch model] was made on a continuous basis: Copenhagen (Georg Rasch and Erling Andersen), Chicago (Benjamin Wright and associates), Australia (David Andrich and associates), [and] Vienna (myself and associates).” These numbers had grown and the Rasch models are presently being used across different fields by different scholars in different parts of the world. In the following section, we provide a brief overview of several influential research clusters identified via DCA.

#### Major Research Clusters Identified by DCA

Different research patterns and themes were evident from the clusters identified. For example, *Cluster#0* focused on measurement invariance in social, behavioral, and health sciences (Engelhard, 2013). Specifically, Rasch models assisted with overcoming several measurement problems, such as the limitations of rating scales (Hobart et al., 2007) and the construction of measures (Wilson, 2005; Bond and Fox, 2007, 2015; Boone et al., 2013). Rasch models were compared with IRT when applied in rating scales analysis (Hobart and Cano, 2009). In the same cluster, some bursts provided guidelines for researchers on how to utilize the advantages of the model (Tennant and Conaghan, 2007) and demonstrated its application in validating the hospital anxiety and depression scale (HADS) total score (HADS-14; Pallant and Tennant, 2007) and the nursing self-efficacy (NSE) scale (Hagquist et al., 2009).

*Cluster#1* emphasized various streams of research on Rasch counterparts, including IRT models (Hambleton et al., 1991; van der Linden and Hambleton, 1997; Baker and Kim, 2004; de Boeck and Wilson, 2004), item and person fit computation in IRT (Glas and Verhelst, 1995; Meijer and Sijtsma, 2001), polytomous IRT models (Embretson and Reise, 2000), and the development of IRT models such as the generalized linear logistic test model (GLLTM) (Patz and Junker, 1999), an extension of the IRT model to overcome problems such as missing data and rated responses, and the multidimensional random coefficients multinomial logit model (Adams et al., 1997).

*Cluster#2* had two primary themes: the unidimensionality requirement in Rasch measurement (e.g., Smith, 2002; Tennant et al., 2004a) and the application of Rasch measurement for validation of instruments that use Likert-type scales in medical fields (Massof and Fletcher, 2001; Massof, 2002; Pesudovs, 2006), such as patient-reported outcome measures (Pesudovs et al., 2007), the Quality of Life Impact of Refractive Correction (QIRC) questionnaire (Pesudovs et al., 2004), the Activities of Daily Vision Scale (ADVS; Pesudovs et al., 2003), and the Impact of Vision Impairment scale (Lamoureux et al., 2006). Finally, *clusters#4* and *#5* centered on the development of two forms of Rasch measurement: latent class Rasch measurement or the mixture Rasch model (e.g., Rost, 1991; see chapters in Fischer and Molenaar, 1995; von Davier, 1996) and many-facet Rasch measurement (e.g., Linacre, 1989; Eckes, 2015).

Based on our examination of the bursts' contents and personal communications with the identified scholars, we propose three groups of hypothetical factors that we view as facilitators of publication and author burstness, rendering three hypotheses. The first hypothesis is related to the publications' educational content and/or the perspectives that the authors on Rasch models have taken in their publications. Influential publications by Bond and Fox (2007, 2015), Engelhard (2013), Tennant and Conaghan (2007), and Boone et al. (2013), for example, are suitable for educators and medical practitioners who need to adopt rigorous measurement methods in their research, a contributing factor of their high citations and bursts. Engelhard (2019, personal communication) stated “I believe that my research is being cited because I write as a teacher […] Measurement is viewed as complex and statistical, while I view measurement as essentially a facet of clear thinking about the constructs in our theories […] I have tried to […] introduce the use of meaningful and invariant scales in numerous fields.” This resonated with Bond's (2019, personal communication) idea about the success of Bond and Fox's (2001, 2007, 2015) book, stressing that making an attempt to communicate the properties of the Rasch model to the ever-growing field of psychology, medicine, and social sciences is a key factor in attracting more scholars to this field. Similarly, Boone (2019, personal communication) highlighted his endeavors to find efficient ways “how to explain Rasch and how to encourage the use of Rasch among non-psychometricians.”

The second hypothesis about bursts concerns the timeliness of publications and the outreach and reputation of authors and publishers. According to Tennant (2019, personal communication), “the impact of [his] publication was a mix of publishing at a time when the use of the Rasch model was expanding rapidly […], the fact that it was written as a teaching paper suitable for clinicians, and the dissemination through Arthritis & Rheumatism-Arthritis Care & Research to the potentially large musculoskeletal community. It is also referenced in our Psychometric Laboratory teaching programme, which is undertaken in many European countries, to a wide range of professionals in health care.”

The third hypothesis exclusively relates to the available Rasch software. Two of the highest bursts were Linacre's *Winsteps* and *Facets*. There are several possible reasons for the status of these Rasch model packages, such as longevity (*Winsteps* and *Facets* started in 1998 and 1987 respectively), comprehensiveness of the outputs and computations, capacity for computation, robustness to missing data, and being instructive (having detailed manuals), and offering prompt support^7^ (Linacre, 2019, personal communication). We call for further research to shed light on the socio-cultural and ethnographic aspects of Rasch measurement research.

### Limitations

The present study is not without its limitations. First, the co-citation method was only able to identify impactful publications and authors only after a sufficient amount of time from their emergence. Therefore, it was incapable of predicting the future of recently published works or publishing authors and, in our view, should not be used to evaluate recent publications. Second, the results were data-driven and accurate only when comprehensive databases were used. It might seem puzzling that Georg Rasch was not among the top 10 influential authors. One reason could be that Rasch did not publish most of his ideas in peer-reviewed journals or books. Accordingly, there was seemingly insufficient acknowledgment of Georg Rasch's role in pioneering the field.

Relatedly, only data from WoS were used in this study; data from other databases such as PubMed and PsyInfo were not used. According to Falagas et al. (2008), PubMed also provides up-to-date articles with early online articles available for access, specifically for authors who are investigating the uses of this database for medical purposes. In our view, WoS is better for investigating Rasch models given its wider coverage and scope compared with other available databases. Future research could compare PubMed and PsyInfo with WoS for investigating Rasch modeling or other research topics and decide on the more comprehensive database to use.

Although comprehensive databases such as the WoS are beneficial for generating the types of data shown in this paper, they may be casting a wide net. For example, one of the most influential authors identified was Hu LT and one of the most influential publications was on structural equation modeling (SEM) (Hu and Bentler, 1999; cited 31,582 times on WoS). The paper mainly compared various fit indexes for SEM analysis. A search for “Rasch” in the documents that cited this article produced 199 hits. These citing articles mainly used Rasch models alongside SEM and cited Hu and Bentler (1999) for use of the fit indices and the criterion provided in SEM analysis (e.g., Rowe et al., 2017; Finbråten et al., 2018; Mairesse et al., 2019). Although the work Hu and Bentler (1999) did not just focus on Rasch models, it had exerted a cross-disciplinary influence on Rasch-related research through its application in Rasch-SEM studies. This finding has two mutually exclusive implications for future research. First, based on this finding, we anticipate that measureable interdisciplinary connections may exist between Rasch measurement and IRT on the one hand and SEM research on the other hand. For example, increasingly more researchers adopt Rasch measurement to validate their data before submitting them to SEM analysis (see Bond and Fox, 2015, p. 240–241). Instead of being quantitatively distinct from each other, these research areas may be interwoven networks with high homogeneity. Alternatively, future researchers who aim for high precision can consider using more stringent keyword searches to reduce the likelihood of including cross-disciplinary publications in the dataset (e.g., “Rasch measurement” AND NOT “structural equation modeling”), although one must be cautious to achieve a good balance between stringent criteria and over-excluding. Having to decide whether to retain the publications that were used in SEM and Rasch measurement or remove them from the analysis is a challenge.

Additionally, the labeling of clusters involved identifying specialties and interpreting the nature of these specialties. Although rewarding and insightful, manual labeling, is tedious and time-consuming. Thus, automatic labeling using algorithms were used in this study, not only to increase the overall efficiency of labeling clusters but also reduce biases (Chen, 2017). However, the labels were limited to the vocabulary used in the data source. This is a key limitation of automatic labeling as the most suitable labels may not exist in the data pool. Using numerous data sources to increase the vocabulary pool may be beneficial in overcoming this limitation. It is important that researchers are in control of the labeling process even when automatic labeling is used (see Aryadoust and Ang, 2019; Aryadoust et al., 2020).

Lastly, only the names of the principal (first) authors were used in the co-citation analyses performed in this study. Databases of cited publications downloaded from WoS did not include the names of other contributing authors even though citing publications did not possess such restriction. If additional author names were made available by these databases, the co-citation analysis may yield different results.

## Conclusion

In this study, we identified research clusters, authors, journals, and keywords that had significant impacts on Rasch measurement research. Using a dual-map overlay, we also revealed multiple inter-domain connections between journals and scientific fields. Informed by personal communications with some of the highly cited authors, we proposed three hypotheses that considered the ethnographic and sociocultural factors to provide a preliminary explanation for the findings. Further research is needed to investigate the relevance of these hypotheses. As the Rasch model required unidimensionality, data-model fit, and local independence, future research may investigate how influential publications on Rasch model are in explaining these concepts and how citing authors conceptualized and applied these concepts. Lastly, this paper took a holistic approach to co-citation analysis of the Rasch measurement field and did not separate different sub-fields. It may be useful to conduct Scientometric studies in specialized fields where Rasch measurement was used for item and person calibration and assessment validation. This may provide in-depth information regarding the status of Rasch measurement in various fields. We hope that the findings of the present study will lead to better understanding of the Rasch measurement frontier.

## Author Contributions

VA conceived the study, created the dataset, conducted the co-citation analysis, and contributed to writing the paper. HT contributed to the literature review and writing the paper. LN contributed to the literature review and revising the paper.

### Conflict of Interest

The authors declare that the research was conducted in the absence of any commercial or financial relationships that could be construed as a potential conflict of interest.

## Footnotes

^1^Andersen (1995) stated that, earlier, Rasch (1961) had suggested a model for polytomous data in Berkeley.

^2^According to Fischer (1995, p. 132), multifactorial Rasch models were initially suggested by Rasch (1965) and later by Micko (1969, 1970) and “have been developed explicitly by Scheiblechner (1971) and Kempf (1972),” as well.

^3^It appeared that Rasch was not against the concept of multidimensionality for person and item parameters. In his presentation on a polytomous model, Rasch (1961) wrote that his model could be extended to multidimensional item/person parameters (Andersen, 1995, p. 272).

^4^See Fischer (1974) for an extended introduction to test theory with an appendix of several improved computer programs for Rasch measurement.

^5^Although Rasch first published his model in 1961, our literature search started in 1972. Unfortunately, neither WoS nor Scopus has indexed any publications by Rasch between 1961 and 1972. Nevertheless, since WoS provided a more comprehensive database of Rasch measurement publications after 1972, the decision was made to use WoS in the present study.

^6^It should be noted that citing articles are not subject to this limitation.

^7^An example is a recent change to *Winsteps* that included increasing the limit on the rating scale from 255 to 32,000 for each item. This was at the request of a user whose data were percentages with two decimal places. This is equivalent to 10001 rating scale categories (Linacre, 2019, personal communication). In addition, Dr Linacre has made a time-sensitive full version of Winsteps available to educators and scholars who plan to teach Rasch model workshops.
