# Supplementary material for: A Scientometric Review of Rasch Measurement: The Rise and Progress of a Specialty
Source: Front Psychol. 2019 Oct 22;10:2197. doi: 10.3389/fpsyg.2019.02197 (PMC6817464; doi:10.3389/fpsyg.2019.02197)
Supplement: Supplementary file 1 [file Data_Sheet_1.pdf]

frontiers in Psychology

A Scientometric Review of Rasch Measurement: The Rise and Progress of a Specialty

Vahid Aryadoust [1]*, Hannah Ann Hui Tan[2] and Li Ying Ng[1]
[1] National Institute of Education, Nanyang Technological University, Singapore, Singapore, [2] School of Social Sciences - Psychology, Nanyang Technological University, Singapore, Singapore

Citation:
Aryadoust, V., Tan, H.A.H., & Ng L.Y. (2019). A Scientometric review of Rasch measurement: The rise and progress of a specialty. *Frontiers in Psychology*. 10:2197. doi: 10.3389/fpsyg.2019.02197

Appendix 1
Author co-citation analysis (ACA)

Cited Authors	Strength	Begin	End	Span
RASCH G	5.4145	1976	1987	11
LORD FM	31.2723	1977	2004	27
WRIGHT BD	7.683	1977	1989	12
HAMBLETON RK	6.1043	1978	1983	5
WHITELY SE	4.8999	1978	1997	19
GUSTAFSSON JE	24.8724	1979	2001	22
MELLENBERGH GJ	6.0442	1981	1998	17
HABERMAN SJ	13.3056	1981	1998	17
SAMEJIMA F	29.5008	1982	2006	24
FORMANN AK	8.3337	1983	1999	16
BOCK RD	32.2103	1983	2007	24
YEN WM	7.4321	1986	2007	21
MOLENAAR IW	30.1958	1986	2005	19
DIVGI DR	5.5671	1986	2002	16
ROSKAM EE	6.5896	1988	1999	11
MISLEVY RJ	24.9837	1988	2007	19
JANSEN PGW	6.3383	1988	1996	8
KELDERMAN H	32.5274	1988	2005	17
BISHOP YM	8.8755	1988	1996	8
VANDENWOLLENBERG AL	10.32	1988	2001	13
DEMPSTER AP	7.3827	1990	2006	16
GUTTMAN L	19.2308	1990	2007	17
ROSENBAUM PR	10.7626	1990	2005	15
GLAS CAW	24.1748	1990	2007	17
MOKKEN RJ	10.7626	1990	2005	15
LAZARSFELD PF	8.8039	1990	2005	15

Cited Authors	Strength	Begin	End	Span
LEHMANN EL	4.8998	1991	2003	12
GOODMAN LA	7.8502	1991	1999	8
KLAUER KC	7.079	1991	2003	12
CHOPPIN B	4.4694	1991	1996	5
DUNCAN OD	9.176	1991	1995	4
CLOGG CC	6.7746	1991	1998	7
CRESSIE N	8.0066	1991	1994	3
TJUR T	10.0266	1991	1997	6
AGRESTI A	16.0409	1992	2000	8
JANNARONE RJ	7.2044	1993	2003	10
FISHER AG	14.1181	1993	1999	6
FOLLMANN D	4.3777	1993	1998	5
LUNZ ME	13.2691	1993	2006	13
HULIN CL	7.0506	1993	2001	8
SILVERSTEIN B	17.1957	1993	2003	10
LINDSAY B	11.1675	1993	2001	8
DELEEUW J	5.6291	1993	1998	5
GRANGER CV	19.9744	1993	2003	10
RAMSAY JO	6.1342	1993	1999	6
VERHELST ND	20.655	1993	2007	14
HEINEMANN AW	14.6612	1993	2002	9
MCCULLAGH P	4.4323	1993	2003	10
DRASGOW F	11.0877	1993	2003	10
HOIJTINK H	15.291	1994	2005	11
MURAKI E	22.8866	1994	2007	13
TATSUOKA KK	4.2288	1995	2001	6
MCARTHUR DL	5.2221	1995	1997	2
SIJTSMA K	5.4376	1995	2001	6
MAHONEY FI	6.1136	1995	2008	13
RECKASE MD	6.6466	1995	2001	6
HATTIE J	5.418	1996	2002	6
ZHU WM	4.3118	1996	2001	5
HAMILTON BB	4.5777	1996	1998	2
JETTE AM	4.8157	1996	2002	6
*SAS I	5.2966	1996	2005	9
GRIMBY G	10.051	1996	2004	8
FRIES JF	6.1401	1996	2004	8
STUCKI G	11.3091	1997	2009	12
MCDONALD RP	13.1225	1997	2005	8
BERGNER M	8.3931	1997	2003	6
ANDERSEN EB	10.5197	1998	2002	4
FISCHER GH	15.771	1998	2004	6
ROST J	15.3905	1998	2006	8
GELMAN A	9.1748	1998	2003	5
ANGOFF WH	10.0569	1998	2005	7

Cited Authors	Strength	Begin	End	Span
PATZ RJ	11.4501	1999	2007	8
MCDOWELL I	5.8817	2000	2002	2
EFRON B	8.72	2000	2005	5
PATRICK DL	6.9765	2000	2003	3
SHROUT PE	5.0793	2001	2006	5
PRIETO L	9.109	2002	2005	3
VAN DER L	9.1301	2002	2006	4
MEIJER RR	8.3293	2002	2005	3
MCHORNEY CA	19.5839	2002	2009	7
RACZEK AE	5.7202	2002	2004	2
ADAMS RJ	9.8462	2002	2006	4
WOLFE F	13.4246	2002	2008	6
WU ML	18.9951	2002	2006	4
WRIGHT B	7.9454	2003	2008	5
MICHELL J	9.3	2003	2006	3
VELOZO CA	29.2273	2004	2009	5
HAYS RD	11.3137	2004	2012	8
MASSOF RW	21.209	2004	2013	9
LAI JS	13.1848	2005	2009	4
TESIO L	13.9526	2005	2013	8
THISSEN D	9.28	2005	2011	6
MERBITZ C	8.2863	2006	2008	2
FOLSTEIN MF	15.5944	2007	2012	5
DE BP	10.3586	2007	2011	4
PESUDOVS K	14.8978	2007	2012	5
SMITH RM	8.0182	2008	2009	1
SMITH E	6.9747	2008	2010	2
KARABATSOS G	12.4787	2008	2009	1
DUNCAN PW	11.4908	2008	2010	2
LAMOUREUX EL	31.5968	2008	2013	5
MALLINSON T	19.2678	2009	2011	2
LUCE RD	12.4377	2009	2013	4
LORD F	12.3599	2009	2011	2
CONRAD KJ	11.7285	2010	2013	3
WAINER H	8.8562	2010	2012	2
LUNDSTROM M	12.7509	2010	2011	1
BAKER FB	10.4419	2010	2012	2
HALEY SM	4.1974	2010	2011	1
BIRNBAUM A	6.0967	2010	2011	1
MANGIONE CM	12.6597	2011	2012	1
PALLANT JF	18.1432	2012	2015	3
**AMERICAN PSYCHIATRIC ASSOCIATION	13.6993	2012	2014	2
WANG WC	27.9064	2012	2016	4
HAGQUIST C	30.5041	2012	2019	7

Cited Authors	Strength	Begin	End	Span
HOBART J	28.1438	2012	2019	7
HU LT	31.2241	2013	2019	6
HOBART JC	23.8062	2013	2015	2
REEVE BB	13.3597	2013	2015	2
[ANONYMOUS]	33.3255	2013	2019	6
MARAIS IDA	14.0817	2014	2015	1
REISE SP	5.3903	2014	2015	1
LINCARE MJ	31.7059	2014	2019	5
MESSICK S	30.2655	2014	2019	5
BEATON DE	12.6599	2014	2015	1
MUTHEN LK	21.8076	2015	2017	2
WOLFE EW	11.112	2015	2019	4
COHEN J	7.4084	2015	2016	1
R CT	26.2344	2015	2019	4
SMITH AB	6.558	2015	2016	1
DEVELLIS RF	15.6989	2016	2019	3
BOONE WJ	43.9175	2016	2019	3
GOTHWAL VK	6.2344	2016	2017	1
DE AYALA R	10.2676	2016	2019	3
KHADKA J	26.1945	2016	2019	3
CHRISTENSEN KB	30.9013	2016	2019	3
ENGELHARD G	42.3541	2016	2019	3
FISHERWP	21.3046	2017	2019	2
TERWEE CB	20.0977	2017	2019	2
MOKKINK LB	24.6906	2017	2019	2
ECKES T	12.33	2017	2019	2

Appendix 2
Document co-citation analysis (DCA)

References (DCA)	Year	Strength	Begin	End	Span
BOND TG, 2007, APPL RASCH MODEL FUN	2007	133.0131	2010	2015	5
BOND TG, 2001, APPL RASCH MODEL FUN	2001	88.1181	2003	2009	6
TENNANT A, 2007, ARTHRIT RHEUM-ARTHR, V57, P1358	2007	83.7317	2011	2015	4
BOND TG, 2015, APPL RASCH MODEL FUN	2015	57.9267	2016	2019	3
PALLANT JF, 2007, BRIT J CLIN PSYCHOL, V46, P1	2007	52.4344	2009	2015	6
SMITH E, 2002, J APPL MEAS, V3, P205	2002	49.3961	2006	2010	4
EMBRETSON SE, 2000, ITEM RESPONSE THEORY	2000	33.1571	2003	2008	5
HAGQUIST C, 2009, INT J NURS STUD, V46, P380, DOI	2009	32.1003	2012	2017	5
ENGELHARD G, 2013, INVARIANT MEASUREMENT: USING RASCH MODELS IN THE SOCIAL	2013	30.8451	2016	2019	3
LINACRE JM, 2002, J APPL MEAS, V3, P85	2002	28.0657	2006	2010	4
HOBART J, 2009, HEALTH TECHNOL ASSES, V13, P1, DOI	2009	27.8369	2012	2017	5
TENNANT A, 2006, RASCH MEAS T, V20, P1048	2006	27.8189	2008	2014	6
TENNANT A, 2004, VALUE HEALTH, V7, P0, DOI	2004	27.3566	2006	2012	6
BOONE WJ, 2014, RASCH ANAL HUMAN SCI	2014	26.274	2016	2019	3
**WORLDHEALTHORGANIZATION, 2001, INT CLASS FUNCT DIS	2001	25.7576	2004	2009	5
TESIO L, 2003, J REHABIL MED, V35, P105, DOI	2003	25.2808	2005	2011	6

References (DCA)	Year	Strength	Begin	End	Span
BOND TG, 2001, APPLYING RASCH MODEL	2001	24.8066	2004	2009	5
PESUDOVS K, 2007, OPTOMETRY VISION SCI, V84, P663, DOI	2007	23.5194	2009	2015	6
WILSON M, 2005, CONSTRUCTING MEASURE	2005	21.3896	2007	2013	6
PESUDOVS K, 2004, OPTOMETRY VISION SCI, V81, P769, DOI	2004	21.2727	2005	2011	6
PESUDOVS K, 2003, INVEST OPHTH VIS SCI, V44, P2892, DOI	2003	21.0882	2004	2011	7
TENNANT A, 2004, MED CARE, V42, P37, DOI	2004	20.8859	2005	2012	7
SMITH AB, 2008, BMC MED RES METHODOL, V8, P0, DOI	2008	20.5055	2011	2016	5
HOBART JC, 2007, LANCET NEUROL, V6, P1094, DOI	2007	20.1648	2010	2015	5
ANDRICH D, 2011, EXPERT REV PHARM OUT, V11, P571	2011	19.9128	2013	2019	6
STREINER DL, 2008, HLTH MEASUREMENT SCA	2008	19.7225	2012	2016	4
MARAIS IDA, 2008, J APPL MEAS, V9, P200	2008	19.1957	2011	2015	4
FISCHER GH, 1995, RASCH MODELS FDN REC	1995	18.6486	1998	2003	5
VAN DER L, 1997, HDB MODERN ITEM RESP	1997	18.5703	1998	2005	7
LINACRE JM, 1999, J OUTCOME MEAS, V3, P103	1999	18.3245	2002	2007	5
LINACRE JM, 2012, USERS GUIDE WINSTEPS	2012	17.9953	2014	2019	5
LAMOUREUX EL, 2006, INVEST OPHTH VIS SCI, V47, P4732, DOI	2006	17.9028	2007	2012	5
SMITH E, 2004, INTRO RASCH MEASUREM	2004	17.3657	2006	2011	5
PESUDOVS K, 2006, BMC OPHTHALMOL, V6, P25, DOI	2006	17.0428	2007	2012	5
HAYS RD, 2000, MED CARE, V38, P28	2000	16.831	2002	2007	5
LINACRE JM, 2009, USERS GUIDE WINSTEPS	2009	16.7212	2010	2017	7
MASSOF RW, 2002, OPTOMETRY VISION SCI, V79, P516, DOI	2002	16.347	2005	2010	5
DE AYALA R, 2009, THEORY PRACTICE ITEM	2009	16.255	2014	2017	3
PETRILLO J, 2015, VALUE HEALTH, V18, P25, DOI	2015	15.8476	2017	2019	2
ANDRICH D, 2003, RUMM 2020	2003	15.8125	2006	2011	5
ANDRICH D, 1988, RASCH MODELS MEASURE	1988	15.5861	1991	1996	5
REEVE BB, 2007, MED CARE, V45, P0, DOI	2007	15.4042	2010	2015	5
MASSOF RW, 2001, VISION RES, V41, P397, DOI	2001	15.0899	2003	2009	6
KHADKA J, 2013, OPTOMETRY VISION SCI, V90, P720, DOI	2013	14.9077	2016	2019	3
DUNCAN PW, 2003, ARCH PHYS MED REHAB, V84, P950, DOI	2003	14.7427	2005	2011	6
SMITH E, 2001, J APPL MEAS, V2, P281	2001	14.5993	2004	2009	5
LINACRE JM, 2005, USERS GUIDE WINSTEPS	2005	14.0175	2008	2011	3
LINACRE JM, 2008, WINSTEPS RASCH MEASU	2008	14.01	2009	2013	4
LINCARE MJ, 2002, RASCH MEASUREMENT T, V16, P878	2002	13.9214	2005	2010	5
MOKKINK LB, 2010, J CLIN EPIDEMIOL, V63, P737, DOI	2010	13.901	2016	2019	3
TERWEE CB, 2007, J CLIN EPIDEMIOL, V60, P34, DOI	2007	13.8813	2013	2015	2
KHADKA J, 2012, HEALTH QUAL LIFE OUT, V10, P0, DOI	2012	13.8624	2016	2019	3
LINACRE JM, 2011, USERS GUIDE WINSTEPS	2011	13.7714	2013	2019	6
MOKKINK LB, 2010, QUAL LIFE RES, V19, P539, DOI	2010	13.6575	2015	2019	4
ADAMS RJ, 1997, APPL PSYCH MEAS, V21, P1, DOI	1997	13.5626	1999	2005	6
MCHORNEY CA, 1997, J CLIN EPIDEMIOL, V50, P451, DOI	1997	13.5626	1999	2005	6
PESUDOVS K, 2010, J CATARACT REFR SURG, V36, P718, DOI	2010	13.5176	2011	2016	5
LINACRE JM, 1998, J OUTCOME MEAS, V2, P266	1998	13.4198	2003	2006	3
CHEN WH, 2014, QUAL LIFE RES, V23, P485, DOI	2014	13.1008	2015	2019	4
HALEY SM, 1994, J CLIN EPIDEMIOL, V47, P671, DOI	1994	12.908	1996	2002	6
NILSSON AL, 2011, J REHABIL MED, V43, P884, DOI	2011	12.4829	2013	2019	6
GARAMENDI E, 2006, VISION RES, V46, P1375, DOI	2006	12.3915	2007	2011	4
WOLFE EW, 2007, J APPL MEAS, V8, P204	2007	12.3871	2011	2015	4
GOTHWAL VK, 2009, OPTOMETRY VISION SCI, V86, P836, DOI	2009	12.1906	2010	2013	3
MERBITZ C, 1989, ARCH PHYS MED REHAB, V70, P308	1989	12.1736	1992	1997	5
SMITH RM, 1998, J OUTCOME MEAS, V2, P66	1998	12.062	2000	2006	6
CHRISTENSEN KB, 2013, RASCH MODELS HLTH	2013	11.9499	2016	2019	3
MCHORNEY CA, 2000, MED CARE, V38, P43	2000	11.8774	2002	2006	4
GRIMBY G, 2012, J REHABIL MED, V44, P97, DOI	2012	11.8552	2015	2019	4
LINACRE J, 2012, WINSTEPS RASCH MEASU	2012	11.7448	2013	2019	6

References (DCA)	Year	Strength	Begin	End	Span
LAI JS, 2003, QUAL LIFE RES, V12, P485, DOI	2003	11.5904	2005	2009	4
CHANG CC, 2015, J CLIN EPIDEMIOL, V68, P674, DOI	2015	11.4286	2016	2019	3
DE BP, 2004, EXPLANATORY ITEM RES	2004	11.305	2007	2012	5
WRIGHT BD, 2000, J APPL MEAS, V1, P83	2000	11.2769	2003	2008	5
HAGQUIST C, 2004, PERS INDIV DIFFER, V36, P955, DOI	2004	11.214	2007	2012	5
FISHERWP, 2007, RASCH MEAS TRANS, V21, P1095	2007	10.8967	2013	2015	2
ANDRICH D, 2012, J EDUC BEHAV STAT, V37, P387, DOI	2012	10.8338	2015	2019	4
GOTHWAL VK, 2009, OPTOMETRY VISION SCI, V86, P1160, DOI	2009	10.8312	2010	2013	3
SMITH RM, 2000, J APPL MEAS, V1, P199	2000	10.8053	2003	2008	5
LAMOUREUX EL, 2008, OPHTHAL EPIDEMIOL, V15, P224, DOI	2008	10.6763	2009	2011	2
KHADKA J, 2011, INVEST OPHTH VIS SCI, V52, P6971, DOI	2011	10.5539	2016	2019	3
ANDRICH D, 2010, RASCH MODELS MEASURE	2010	10.5502	2016	2019	3
MURAKI E, 1992, APPL PSYCH MEAS, V16, P159, DOI	1992	10.524	1995	2000	5
HEINEMANN AW, 1993, ARCH PHYS MED REHAB, V74, P566, DOI	1993	10.524	1995	2000	5
LAMOUREUX EL, 2007, INVEST OPHTH VIS SCI, V48, P1001, DOI	2007	10.4751	2008	2014	6
DA RNS, 2013, J PSYCHIATR RES, V47, P141, DOI	2013	10.3065	2014	2019	5
WRIGHT BD, 1989, ARCH PHYS MED REHAB, V70, P857	1989	10.2467	1992	1997	5
LINACRE JM, 2016, WINSTEPS RASCH MEASU	2016	10.172	2017	2019	2
ANDRICH D, 2004, MED CARE, V42, P7, DOI	2004	10.1361	2008	2012	4
VELOZO CA, 2000, J OUTCOME MEAS, V4, P667	2000	10.0865	2004	2008	4
BOND T, 2007, APPL RASCH MODEL	2007	10.0605	2012	2015	3
MALLINSON T, 2007, OPTOMETRY VISION SCI, V84, P675, DOI	2007	10.0149	2009	2012	3
PATRICK DL, 2011, VALUE HEALTH, V14, P967, DOI	2011	9.9934	2016	2019	3
LINACRE J, 2006, WINSTEPS RASCH MEASU	2006	9.9107	2007	2012	5
VELOZO CA, 1999, AM J OCCUP THER, V53, P83, DOI	1999	9.8793	2002	2007	5
DE BOER M, 2004, OPHTHAL PHYSL OPT, V24, P257, DOI	2004	9.8377	2006	2010	4
FISHER AG, 1993, AM J OCCUP THER, V47, P319, DOI	1993	9.7671	1994	2000	6
MALLINSON T, 2004, MED CARE, V42, P17, DOI	2004	9.6098	2009	2011	2
LUNDSTROM M, 2009, J CATARACT REFR SURG, V35, P504, DOI	2009	9.5608	2009	2011	2
HAMBLETON RK, 1991, FUNDAMENTALS ITEM RE	1991	9.5062	1995	1999	4
MASSOF RW, 2001, SURV OPHTHALMOL, V45, P531, DOI	2001	9.3916	2003	2008	5
SHEA TL, 2009, BMC PSYCHIATRY, V9, P0, DOI	2009	9.3701	2014	2017	3
PATRICK DL, 2011, VALUE HEALTH, V14, P978, DOI	2011	9.2548	2015	2019	4
LAMOUREUX E, 2011, AM J OPHTHALMOL, V151, P195, DOI	2011	9.2438	2013	2019	6
PRIETO L, 2003, HEALTH QUAL LIFE OUTCOMES, V1, P27, DOI	2003	9.2227	2007	2011	4
MARAIS IDA, 2008, J APPL MEAS, V9, P105	2008	9.2013	2014	2016	2
LINACRE JM, 2014, WINSTEPS RASCH MEASU	2014	9.1847	2014	2019	5
LINACRE J, 2009, RASCH MEAS T, V23, P1228	2009	9.1471	2015	2017	2
**AMERICANEDUCATIONRESEARCHASSOCIATIONAMERICANPSYCHOLOGICALASSOCIATION&NATIONALCOUNCILONMEASUREMENTINEDUCATION, 2014, STAND ED PSYCH TEST	2014	9.039	2017	2019	2
ANDRICH D, 2015, EDUC PSYCHOL MEAS, V75, P185, DOI	2015	9.039	2017	2019	2
LINACRE JM, 2016, USERS GUIDE WINSTEPS	2016	9.039	2017	2019	2
CAPPELLERI JC, 2014, CLIN THER, V36, P648, DOI	2014	9.039	2017	2019	2
LINACRE JM, 1994, ARCH PHYS MED REHAB, V75, P127	1994	8.871	1995	1999	4
SILVERSTEIN B, 1992, ARCH PHYS MED REHAB, V73, P507	1992	8.871	1995	1999	4
STUCKI G, 1996, J CLIN EPIDEMIOL, V49, P711, DOI	1996	8.7492	1999	2004	5
DEVELLIS RF, 2012, SCALE DEV THEORY APP	2012	8.7105	2015	2016	1
R CT, 2013, R LANG ENV STAT COMP	2013	8.5702	2015	2019	4
LINACRE JM, 2006, USERS GUIDE WINSTEPS	2006	8.5367	2008	2014	6
STREINER D, 2003, HLTH MEASUREMENT SCA	2003	8.4923	2006	2010	4
FORKMANN T, 2009, REHABIL PSYCHOL, V54, P186, DOI	2009	8.4437	2013	2014	1
BOONE WJ, 2011, SCI EDUC, V95, P258, DOI	2011	8.4305	2014	2017	3
PESUDOVS K, 2010, OPTOMETRY VISION SCI, V87, P285, DOI	2010	8.2228	2011	2016	5

References (DCA)	Year	Strength	Begin	End	Span
MANGIONE CM, 2001, ARCH OPHTHALMOL-CHIC, V119, P1050, DOI	2001	8.1752	2005	2008	3
RAICHE G, 2005, RASCH MEASUREMENT T, V19, P1012	2005	8.1466	2012	2013	1
LINDSAY B, 1991, J AM STAT ASSOC, V86, P96, DOI	1991	8.0526	1993	1999	6
PATZ RJ, 1999, J EDUC BEHAV STAT, V24, P146, DOI	1999	8.0104	2001	2007	6
LAMOUREUX EL, 2009, INVEST OPHTH VIS SCI, V50, P2607, DOI	2009	7.9882	2010	2011	1
BRODERSEN J, 2007, J MED ECON, V10, P309, DOI	2007	7.9176	2013	2015	2
CHANG KC, 2014, HEALTH QUAL LIFE OUT, V12, P0, DOI	2014	7.9067	2017	2019	2
BELVEDERE SL, 2010, J CLIN EPIDEMIOL, V63, P1287, DOI	2010	7.8674	2012	2015	3
ECKES T, 2011, INTRO MANY FACET RAS	2011	7.8111	2014	2016	2
KHAN A, 2013, J CLIN EPIDEMIOL, V66, P681, DOI	2013	7.8088	2017	2019	2
KARABATSOS G, 2001, J APPL MEAS, V2, P389	2001	7.7626	2006	2009	3
LINACRE JM, 2009, WINSTEPS VERSION 3 6	2009	7.6917	2010	2012	2
MCHORNEY CA, 1997, ANN INTERN MED, V127, P743, DOI	1997	7.6532	1999	2004	5
SMITH AB, 2006, PSYCHO-ONCOLOGY, V15, P817, DOI	2006	7.6529	2009	2012	3
GLAS CAW, 1995, RASCH MODELS, V0, P69	1995	7.6392	1999	2003	4
LINACRE JM, 2005, WINSTEPS RASCH MEASU	2005	7.5852	2007	2012	5
NORQUIST JM, 2004, MED CARE, V42, P25, DOI	2004	7.4965	2010	2011	1
WRIGHT BD, 1994, RASCH MEASUREMENT T, V8, P370	1994	7.4643	1997	2001	4
PESUDOVS K, 2010, BRIT J OPHTHALMOL, V94, P777, DOI	2010	7.4641	2011	2012	1
HATZINGER R, 2007, J STAT SOFTW, V20, P9, DOI	2007	7.2371	2014	2015	1
WAUGH RF, 2005, J APPL MEAS, V6, P80	2005	7.2229	2007	2012	5
LUNDGREN-NILSSON A, 2005, J REHABIL MED, V37, P23, DOI	2005	7.2195	2007	2010	3
WOLFE F, 1999, ANN RHEUM DIS, V58, P563, DOI	1999	7.1954	2002	2004	2
SIEGERT RJ, 2010, DISABIL REHABIL, V32, P8, DOI	2010	7.1042	2013	2019	6
RACZEK AE, 1998, J CLIN EPIDEMIOL, V51, P1203, DOI	1998	6.8837	2002	2005	3
FLIEGE H, 2005, QUAL LIFE RES, V14, P2277, DOI	2005	6.8423	2009	2010	1
CHANG WC, 1995, ARCH PHYS MED REHAB, V76, P934, DOI	1995	6.8191	1997	2003	6
STELMACK JA, 2004, INVEST OPHTH VIS SCI, V45, P3919, DOI	2004	6.7547	2006	2007	1
HU LT, 1999, STRUCT EQU MODELING, V6, P1, DOI	1999	6.7547	2006	2007	1
**AMERICANPSYCHIATRICASSOCIATION, 2013, DIAGN STAT MAN MENT	2013	6.6737	2014	2019	5
WHITENECK G, 2009, ARCH PHYS MED REHAB, V90, P0, DOI	2009	6.6623	2016	2017	1
MASSOF RW, 2005, OPHTHAL EPIDEMIOL, V12, P103, DOI	2005	6.6575	2007	2008	1
FISCHER GH, 1995, RASCH MODELS, V0, P15	1995	6.635	2000	2003	3
**NATIONALRESEARCHCOUNCIL, 2012, FRAMEWORK K 12 SCI E	2012	6.6054	2017	2019	2
JAFARI P, 2012, HEALTH QUAL LIFE OUT, V10, P0, DOI	2012	6.6054	2017	2019	2
LINACRE J, 2004, INTRO RASCH MEASUREM, V0, P258, DOI	2004	6.601	2009	2011	2
STELMACK JA, 2002, INVEST OPHTH VIS SCI, V43, P2859	2002	6.5492	2004	2008	4
GELMAN A, 1995, BAYESIAN DATA ANAL	1995	6.462	1999	2003	4
LINACRE JM, 1997, USERS GUIDE BIGSTEPS	1997	6.462	1999	2003	4
TENNANT A, 1996, BRIT J RHEUMATOL, V35, P574	1996	6.3264	1997	2004	7
MEIJER RR, 2001, APPL PSYCH MEAS, V25, P107, DOI	2001	6.3091	2002	2005	3
LINACRE JM, 2008, USERS GUIDE WINSTEPS	2008	6.2735	2010	2011	1
ANDRICH D, 2003, RUMM2020	2003	6.2712	2009	2010	1
CANO SJ, 2011, PATIENT PREFER ADHER, V5, P279, DOI	2011	6.2095	2017	2019	2
BOONE WJ, 2006, SCI EDUC, V90, P253, DOI	2006	6.1882	2013	2014	1
BROWN TA, 2006, CONFIRMATORY FACTOR	2006	6.1882	2013	2014	1
GRIMBY G, 1996, ARCH PHYS MED REHAB, V77, P1109, DOI	1996	6.1835	2000	2004	4
WRIGHT BD, 1979, BEST TEST DESIGN	1979	6.1724	1981	1986	5
BOND TG, 2007, APPLYING RASCH MODEL	2007	6.1505	2010	2012	2
BAKER FB, 2004, ITEM RESPONSE THEORY	2004	6.1367	2007	2012	5
STREINER DL, 1995, HLTH MEASUREMENT SCA	1995	6.0309	2000	2003	3
KANE MT, 2013, J EDUC MEAS, V50, P1, DOI	2013	6.004	2017	2019	2
DAVIS AM, 2003, J CLIN EPIDEMIOL, V56, P1076, DOI	2003	5.8812	2008	2009	1
NORQUIST JM, 2004, MED CARE, V42, P125	2004	5.8812	2008	2009	1

References (DCA)	Year	Strength	Begin	End	Span
FISHER WP, 1995, ARCH PHYS MED REHAB, V76, P113, DOI	1995	5.8736	1999	2003	4
R CT, 2014, R LANG ENV STAT COMP	2014	5.8025	2015	2016	1
MASSOF RW, 2005, ARCH PHYS MED REHAB, V86, P954, DOI	2005	5.7729	2007	2010	3
TURANO KA, 1999, INVEST OPHTH VIS SCI, V40, P865	1999	5.7691	2001	2004	3
LUNZ ME, 1990, APPLIED MEASUREMENT, V3, P331, DOI	1990	5.7633	1994	1998	4
GOTHWAL VK, 2010, J CATARACT REFR SURG, V36, P1181, DOI	2010	5.7506	2011	2014	3
ROST J, 2004, LEHRBUCH TESTTHEORIE	2004	5.7023	2005	2006	1
GOTHWAL VK, 2009, J CATARACT REFR SURG, V35, P1509, DOI	2009	5.7023	2010	2011	1
WOLF J, 2007, PAEDIATR RESPIR REV, V8, P204, DOI	2007	5.7002	2009	2010	1
ANDRICH D, 2003, J APPL MEAS, V4, P205	2003	5.7002	2009	2010	1
PESUDOVS K, 2006, INVEST OPHTH VIS SCI, V47, P2789, DOI	2006	5.7002	2009	2010	1
WARE JE, 1992, MED CARE, V30, P473, DOI	1992	5.6441	1996	2000	4
ELHAN AH, 2008, BMC MUSCULOSKEL DIS, V9, P0, DOI	2008	5.6248	2013	2014	1
EDELEN MO, 2007, QUAL LIFE RES, V16, P5, DOI	2007	5.5645	2014	2015	1
ANDRICH D, 2012, APPL PSYCH MEAS, V36, P309, DOI	2012	5.5645	2014	2015	1
FISCHER GH, 1974, EINFUHRUNG THEORIE P	1974	5.4969	1979	1981	2
WRIGHT BD, 1977, J EDUC MEAS, V14, P97, DOI	1977	5.4969	1979	1981	2
RASCH G, 1980, PROBABILISTIC MODELS	1980	5.4943	1982	1988	6
KELDERMAN H, 1984, PSYCHOMETRIKA, V49, P223, DOI	1984	5.4711	1988	1992	4
MCHORNEY CA, 2004, MED CARE, V42, P73, DOI	2004	5.446	2005	2008	3
PATZ RJ, 1999, J EDUC BEHAV STAT, V24, P342, DOI	1999	5.4262	2002	2007	5
KELDERMAN H, 1994, PSYCHOMETRIKA, V59, P149, DOI	1994	5.4241	1997	2002	5
RYSER L, 1999, ARTHRIT CARE RES, V12, P331, DOI	1999	5.394	2002	2004	2
CELLA D, 2000, MED CARE, V38, P66	2000	5.394	2002	2004	2
LI MNF, 1997, APPL PSYCH MEAS, V21, P215, DOI	1997	5.3609	2000	2005	5
BEATON DE, 2000, SPINE, V25, P3186, DOI	2000	5.3329	2004	2006	2
TENNANT A, 2004, MED CARE S, V42, P137, DOI	2004	5.2922	2008	2009	1
WRIGHT BD, 1996, STRUCT EQU MODELING, V3, P3, DOI	1996	5.2854	1999	2003	4
TESIO L, 1997, PAIN, V69, P269, DOI	1997	5.2854	1999	2003	4
MCARTHUR DL, 1991, ARCH PHYS MED REHAB, V72, P296	1991	5.2428	1995	1997	2
FISHER WP, 1993, AM J OCCUP THER, V47, P331, DOI	1993	5.2428	1995	1997	2
WU ML, 1998, ACER CONQUEST GEN IT	1998	5.1604	2002	2005	3
LINACRE JM, 2009, WINSTEPS RASCH MEASU	2009	5.1239	2010	2012	2
DEMARS C, 2010, ITEM RESPONSE THEORY	2010	5.1056	2014	2016	2
LINACRE J, 2011, WINSTEPS RASCH MEASU	2011	5.0809	2012	2016	4
MARGOLIS MK, 2002, PHARMACOECONOMICS, V20, P791, DOI	2002	5.0355	2004	2008	4
ANDRICH D, 2010, RUMM2030	2010	5.0072	2014	2015	1
TENNANT A, 1996, CLIN REHABIL, V10, P301	1996	4.9464	2003	2004	1
WOLFE F, 2001, J RHEUMATOL, V28, P982	2001	4.9464	2003	2004	1
SVENSSON E, 2001, J REHABIL MED, V33, P47, DOI	2001	4.9219	2004	2007	3
WHITE LJ, 2002, ARCH PHYS MED REHAB, V83, P822, DOI	2002	4.8502	2004	2009	5
HAMBLETON RK, 2000, MED CARE, V38, P60	2000	4.8492	2002	2006	4
ROST J, 1999, PSYCHOL RUNDSCH, V50, P140, DOI	1999	4.824	2000	2005	5
GOTHWAL VK, 2010, INVEST OPHTH VIS SCI, V51, P694, DOI	2010	4.8028	2012	2015	3
PRIETO L, 1998, J OUTCOME MEAS, V2, P285	1998	4.7939	2002	2004	2
ROST J, 1991, BRIT J MATH STAT PSY, V44, P75, DOI	1991	4.7013	1998	1999	1
CONRAD KJ, 2004, MED CARE, V42, P1, DOI	2004	4.6108	2010	2012	2
BAKER FB, 1992, ITEM RESPONSE THEORY	1992	4.5973	1998	2000	2
GOTHWAL VK, 2009, CLIN EXP OPHTHALMOL, V37, P785, DOI	2009	4.5943	2014	2016	2
BRADLOW ET, 1999, PSYCHOMETRIKA, V64, P153, DOI	1999	4.5863	2002	2005	3
LINACRE JM, 1989, MANY FACETED RASCH M	1989	4.5769	1993	1996	3
MYFORD CM, 2003, J APPL MEAS, V4, P386	2003	4.5631	2006	2008	2
MASSOF RW, 2007, OPTOMETRY VISION SCI, V84, P763, DOI	2007	4.541	2012	2014	2
ROSSEEL Y, 2012, J STAT SOFTW, V48, P1	2012	4.5139	2017	2019	2

References (DCA)	Year	Strength	Begin	End	Span
ADAMS RJ, 2012, EDUC PSYCHOL MEAS, V72, P547, DOI	2012	4.5139	2017	2019	2
ZICKAR MJ, 1996, APPL PSYCH MEAS, V20, P71, DOI	1996	4.4624	2000	2002	2
MCDOWELL I, 1996, MEASURING HLTH GUIDE	1996	4.4624	2000	2002	2
RIJMEN F, 2003, PSYCHOL METHODS, V8, P185, DOI	2003	4.4337	2005	2006	1
FOX CM, 1998, J COUNS PSYCHOL, V45, P30	1998	4.4063	2001	2005	4
HALEY SM, 2004, MED CARE, V42, P49, DOI	2004	4.3885	2004	2005	1
TENNANT A, 2007, RASCH MEASUREMENT T, V24, P1082	2007	4.3655	2012	2015	3
ADAMS RJ, 1997, J EDUC BEHAV STAT, V22, P47, DOI	1997	4.3275	2003	2004	1
MASSOF RW, 1998, OPTOMETRY VISION SCI, V75, P349, DOI	1998	4.3237	2001	2003	2
REVICKI DA, 1997, QUAL LIFE RES, V6, P595, DOI	1997	4.2874	2000	2005	5
VELOZO CA, 1995, ARCH PHYS MED REHAB, V76, P705, DOI	1995	4.2197	2000	2003	3
KLAUER KC, 1995, RASCH MODELS, V0, P97	1995	4.2197	2000	2003	3
LINACRE JM, 2014, USERS GUIDE WINSTEPS	2014	4.1469	2016	2019	3
SMITH E, 2000, J APPL MEAS, V1, P303	2000	4.1466	2004	2006	2
ANDERSEN EB, 1973, CONDITIONAL INFERENC	1973	4.1214	1979	1981	2

Appendix 3

Journal co-citation analysis (JCA). This analysis also includes the title of influential books.

Cited Journals (JCA)	Strength	Begin	End	Span
PSYCHOMETRIKA	68.4709	1976	1999	23
J EDUC MEAS	26.9571	1977	2000	23
EDUC PSYCHOL MEAS	4.3176	1977	1981	4
BRIT J MATH STAT PSY	21.2794	1978	2001	23
CONDITIONAL INFERENC	4.137	1979	1981	2
EINFUHRUNG THEORIE P	5.5169	1979	1981	2
REV EDUC RES	4.3638	1980	1999	19
APPLIED PSYCHOL MEAS	6.7869	1980	1992	12
ANN STAT	4.4518	1981	1998	17
BEST TEST DESIGN	6.1911	1981	1986	5
PSYCHOL BULL	13.9553	1981	2003	22
Z EXP ANGEW PSYCHOL	4.1393	1981	1983	2
BIOMETRICS	9.6156	1982	2006	24
PROBABILISTIC MODELS	5.5096	1982	1988	6
APPL PSYCH MEAS	36.9212	1983	2000	17
ANN M AM ED RES ASS	10.6826	1986	2003	17
J EDUC STAT	15.6437	1990	2001	11
RASCH MODELS MEASURE	16.789	1991	1996	5
LATENT TRAIT LATENT	6.7044	1991	1995	4
J AM STAT ASSOC	25.1502	1991	2007	16
BIOMETRIKA	7.0563	1992	2002	10
FACETS COMPUTER PROG	6.4984	1992	1996	4
APPLIED MEASUREMENT	16.8069	1992	2000	8
ED MEASUREMENT	9.9681	1992	1996	4
APR ANN M AM ED RES	4.7643	1992	1994	2
J ROY STAT SOC B MET	4.3683	1993	2000	7
MANY FACETED RASCH M	7.4913	1993	2000	7

AM J OCCUP THER	14.021	1993	2002	9
SOCIOL METHODOL	6.8603	1993	2003	10
ED MEASUREMENT ISSUE	7.7417	1994	2002	8
OBJECTIVE MEASUREMEN	22.5143	1994	2004	10
PHYS MED REH CLIN N	4.441	1995	2000	5
FUNDAMENTALS ITEM RE	10.3875	1995	1999	4
SCAND J REHABIL MED	19.9581	1995	2004	9
AM J PHYS MED REHAB	20.4641	1995	2009	14
PSYCHOL REV	4.6227	1995	1998	3
USERS GUIDE BIGSTEPS	24.4569	1995	2005	10
METHODIKA	4.9443	1995	2001	6
RES Q EXERCISE SPORT	4.3735	1996	2001	5
RASCH MODELS	33.6824	1996	2003	7
BIGSTEPS	6.4137	1996	2000	4
J AM SOC INFORM SCI	4.4885	1996	2000	4
ACTA NEUROL SCAND	5.199	1997	2007	10
AM J PUBLIC HEALTH	6.1733	1997	2002	5
QUALITY LIFE PHARMAC	8.55	1997	2004	7
BRIT J RHEUMATOL	14.8325	1997	2004	7
ANN INTERN MED	13.6894	1997	2004	7
PAIN	10.6173	1997	2009	12
APPL LATENT TRAIT LA	15.575	1998	2005	7
RASCH MODELS FDN REC	23.1257	1998	2003	5
J OUTCOME MEAS	74.2638	1998	2007	9
COMPUTERIZED ADAPTIV	10.0382	1998	2006	8
CONQUEST GEN ITEM RE	5.0698	1999	2005	6
DIAGNOSTICA	11.262	1999	2006	7
LEHRBUCH TESTTHEORIE	7.504	1999	2006	7
J PERS SOC PSYCHOL	9.114	1999	2006	7
BAYESIAN DATA ANAL	6.767	1999	2003	4
BRIT J EDUC PSYCHOL	4.4368	1999	2002	3
STRUCT EQU MODELING	15.6822	1999	2007	8
MEASURING HLTH GUIDE	4.5182	2000	2002	2
J GERONTOL B-PSYCHOL	7.5222	2000	2003	3
J GERONTOL A-BIOL	8.3084	2000	2008	8
ARTHRIT CARE RES	8.6519	2000	2004	4
PSYCHOL RUNDSCH	4.5182	2000	2002	2
PHARMACOECONOMICS	5.9805	2000	2004	4
PSYCHOMETRIC THEORY	5.164	2000	2002	2
ARTHRITIS RHEUM	9.233	2000	2008	8
METHODS PSYCHOL RES	5.3821	2000	2004	4
J APPL PSYCHOL	4.4867	2001	2003	2
ESSAYS ITEM RESPONSE	5.7695	2001	2003	2
J EDUC BEHAV STAT	14.839	2001	2007	6
J HEAD TRAUMA REHAB	6.9399	2002	2006	4
ADV MEASUREMENT ED R	6.9399	2002	2006	4

ED RES METHODOLOGY M	4.2394	2002	2005	3
MULTIVAR BEHAV RES	7.5412	2002	2007	5
STROKE	13.1618	2002	2009	7
CLIN REHABIL	13.52	2002	2011	9
J RHEUMATOL	13.9292	2002	2010	8
ITEM RESPONSE THEORY	40.6607	2003	2008	5
BRIT MED J	11.2386	2003	2010	7
RHEUMATOLOGY	14.2947	2003	2010	7
PSYCHOL METHODS	19.5114	2003	2007	4
APPLYING RASCH MODEL	32.4572	2003	2009	6
WINSTEPS RASCH ANAL	4.9837	2003	2005	2
SURV OPHTHALMOL	7.9516	2004	2007	3
ARCH OPHTHALMOL-CHIC	19.2096	2004	2013	9
VISION RES	26.6855	2004	2010	6
INT CLASS FUNCT DIS	29.497	2004	2009	5
MED CARE	59.4932	2004	2012	8
STAT MED	16.6398	2004	2008	4
SPINE	17.6462	2004	2009	5
PERS INDIV DIFFER	6.3753	2005	2012	7
J APPL MEAS	62.4672	2005	2010	5
J NEUROL NEUROSUR PS	9.9743	2006	2014	8
INTRO RASCH MEASUREM	44.6339	2006	2012	6
OPHTHAL EPIDEMIOL	14.6407	2007	2013	6
ANN RHEUM DIS	18.5242	2007	2012	5
EXPLANATORY ITEM RES	9.0662	2007	2011	4
HEALTH QUAL LIFE OUTCOMES	23.0979	2007	2012	5
J AM GERIATR SOC	20.6708	2007	2011	4
OPHTHAL PHYSL OPT	10.5338	2007	2010	3
RUMM 2020	14.5555	2007	2010	3
BMC OPHTHALMOL	14.8401	2008	2011	3
RASCH MEAS T	21.0534	2009	2014	5
ARTHRIT RHEUM-ARTHR	80.6204	2009	2015	6
REHABIL PSYCHOL	20.2173	2009	2013	4
BRIT J CLIN PSYCHOL	49.9896	2009	2015	6
APPL MEAS EDUC	7.073	2009	2010	1
BRAIN INJURY	19.4561	2009	2012	3
DEV MED CHILD NEUROL	7.8956	2009	2012	3
NEUROLOGY	12.9825	2010	2013	3
BRIT J OPHTHALMOL	7.197	2010	2013	3
CONSTRUCTING MEASURE	21.6576	2010	2013	3
INT J GERIATR PSYCH	10.6283	2010	2012	2
J CATARACT REFR SURG	12.5145	2010	2016	6
STAT SOC BEHAV SC	13.8797	2011	2012	1
BMC MED RES METHODOL	22.1942	2012	2016	4
HEALTH TECHNOL ASSES	31.3782	2012	2017	5
LANCET NEUROL	22.6765	2012	2015	3

INT J NURS STUD	32.0875	2012	2017	5
NEW ENGL J MED	16.2801	2013	2014	1
SOC SCI MED	11.7992	2013	2014	1
PSYCHO-ONCOLOGY	14.9032	2013	2014	1
AM J OPHTHALMOL	5.1204	2013	2014	1
BMC MUSCULOSKEL DIS	17.5562	2013	2017	4
HLTH MEASUREMENT SCA	15.7046	2013	2015	2
J AFFECT DISORDERS	17.072	2014	2017	3
PHYS THER	8.7087	2014	2016	2
R LANG ENV STAT COMP	32.0218	2014	2019	5
PLOS ONE	83.7516	2015	2019	4
EXPERT REV PHARM OUT	9.4094	2015	2016	1
J PSYCHOSOM RES	9.888	2015	2017	2
BMC PUBLIC HEALTH	23.8413	2015	2019	4
JAMA-J AM MED ASSOC	10.0844	2015	2017	2
BMC HEALTH SERV RES	11.4836	2015	2019	4
J RES SCI TEACH	21.0856	2016	2019	3
HEALTH QUAL LIFE OUT	43.4422	2016	2019	3
LANG TEST	26.6721	2016	2019	3
LANCET	15.578	2016	2019	3
OPHTHALMOLOGY	6.5422	2016	2017	1
SCI EDUC	17.9859	2016	2019	3
THESIS	53.7951	2016	2019	3
INT J SCI EDUC	20.1118	2016	2019	3
RASCH ANAL HUMAN SCI	31.7571	2016	2019	3
INVARIANT MEASUREMENT: USING RASCH MODELS IN THE SOCIAL	28.0581	2016	2019	3
ASSESSMENT	26.7557	2016	2019	3
PSYCHOL ASSESSMENT	5.8113	2017	2019	2
J STAT SOFTW	12.7044	2017	2019	2
FRONT PSYCHOL	24.7431	2017	2019	2
PROCD SOC BEHV	21.5745	2017	2019	2
RASCH MODELS HLTH	24.2148	2017	2019	2
